# Development of a 3D Printed Coating Shell to Control the Drug Release of Encapsulated Immediate-Release Tablets

**DOI:** 10.3390/polym12061395

**Published:** 2020-06-22

**Authors:** Mohammed S. Algahtani, Abdul Aleem Mohammed, Javed Ahmad, Ehab Saleh

**Affiliations:** 1 Department of Pharmaceutics, College of Pharmacy, Najran University, Najran 66433, Kingdom of Saudi Arabia; aaleem@nu.edu.sa (A.A.M.); jaahmed@nu.edu.sa (J.A.); 2 Future Manufacturing Processes Research Group, Faculty of Engineering and Physical Sciences, University of Leeds, Leeds LS2 9JT, UK; E.Saleh@leeds.ac.uk

**Keywords:** 3D printing technology, solid dosage forms, controlled release, propranolol HCl, cellulose acetate, release kinetic, personalized medicine

## Abstract

The use of 3D printing techniques to control drug release has flourished in the past decade, although there is no generic solution that can be applied to the full range of drugs or solid dosage forms. The present study provides a new concept, using the 3D printing technique to print a coating system in the form of shells with various designs to control/modify drug release in immediate-release tablets. A coating system of cellulose acetate in the form of an encapsulating shell was printed through extrusion-based 3D printing technology, where an immediate-release propranolol HCl tablet was placed inside to achieve a sustained drug release profile. The current work investigated the influence of shell composition by using different excipients and also by exploring the impact of shell size on the drug release from the encapsulated tablet. Three-dimensional printed shells with different ratios of rate-controlling polymer (cellulose acetate) and pore-forming agent (D-mannitol) showed the ability to control the amount and the rate of propranolol HCl release from the encapsulated tablet model. The shell-print approach also showed that space/gap available for drug dissolution between the shell wall and the enclosed tablet significantly influenced the release of propranolol HCl. The modified release profile of propranolol HCl achieved through enclosing the tablet in a 3D printed controlled-release shell followed Korsmeyer–Peppas kinetics with non-Fickian diffusion. This approach could be utilized to tailor the release profile of a Biopharmaceutics Classification System (BCS) class I drug tablet (characterized by high solubility and high permeability) to improve patient compliance and promote personalized medicine.

## 1. Introduction

The concept of a *one-size-fits-all* dosage form is challenging and difficult to achieve due to the high individual variability related to genetics, ethnicity, gender, age, and patient weight [1]. In real practice, some patients will be exposed to either high or low doses, with exposure to unwanted side effects. The concept of personalized medicine has grown dramatically in popularity in recent years, as dose adjustments can be made according to the pharmacokinetic and pharmacogenetic profile of the patient [2].

Three-dimensional printing technologies have been exploited for personalized drug delivery [3]. To achieve the goal of personalized medicine, the 3D printing technique was utilized to design, for example, a five-in-one dose combination polypill with defined immediate and extended-release profiles [4], a multi-layered polypill as a platform for multi-drug therapy production [5], tablets with fully customizable release profiles [6], and dosage forms to deliver tailored individualized precision dosing of anti-coagulant [7]. This technology can be used to fabricate pharmaceutical formulations in different sizes and shapes using a variety of materials with customized drug concentrations and release profiles that cannot be produced using conventional mass production methods in the pharmaceutical industry [8]. Different types of 3D printers using inkjet printing [9], stereolithography (SLA) [10], selective laser sintering (SLS) [11], fused deposition modeling [12], and extrusion-based pressure-assisted micro syringes [13] have been used to develop various types of drug delivery systems and novel devices such as transdermal patches [14], intrauterine and sub-cutaneous devices [15,16], and biodegradable implants [17]. Recently, Dumpa et al. developed a gastro-retentive floating pulsatile drug delivery system where the investigator enclosed a theophylline tablet prepared by direct compression into a floating shell, exploiting the 3D printing technique (hot-melt extrusion-paired fused-deposition modeling) for the treatment of chronic asthma [18].

Here, we propose a potential application of 3D printing technology to modify the release profile of an immediate-release tablet. An extrusion-based printing method was used in this work as it allows for a broad selection of suitable polymers and excipients, and also allows for printing with different polymer and excipients ratios; therefore, modified release profiles could be easily achieved by printing solid dosage forms of specific desirability, exploiting the computer-aided design (CAD) process. The current study aims to develop an encapsulating system (shell) to provide a controlled-release coating layer over an immediate-release tablet (using propranolol HCl (Indicardin^®^, 40 mg tablet) as a model) to achieve a sustained release profile of the drug. Propranolol HCl is a beta-blocker commercially available in different dosage forms, including capsules, tablets, and oral solutions. Propranolol HCl has a short elimination half-life (3 h). Therefore, frequent dosing (3–4 times a day) is prescribed to achieve optimal therapeutic efficacy. The development of a drug delivery system to deliver propranolol HCl at a controlled rate is required to minimize the dosing frequency and reduce fluctuations in systemic drug concentrations [19]. Based on the Biopharmaceutics Classification System (BCS), propranolol HCl is a class I drug (high soluble and high permeable), with a dose number (Do) of 0.0025. These characteristics of propranolol HCl make it an excellent candidate for the development of modified-release dosage products [20]. The tablet was placed inside a 3D printed controlled-release shell to observe the change in dissolution profile and release kinetics of propranolol HCl.

## 2. Materials and Methods

### 2.1. Materials

Propranolol HCl (Indicardin^®^, 40 mg) was supplied by the Najran University Hospital pharmacy, Najran, Saudi Arabia. Cellulose acetate, D-mannitol, and polyethylene glycol (PEG) 6000 were purchased from Sigma-Aldrich (Gillingham, UK). Ethanol, acetone, dimethyl sulfoxide (DMSO) were purchased from UFC Biotech (Riyadh, KSA), and all other reagents were of analytical grade.

### 2.2. Preparation of Material for Extrusion-Based 3D Printing of Controlled-Release Tablet Shells

A hydrophobic polymer was selected to prepare 3D printed controlled-release shells in order to modify the release of enclosed immediate release tablet. Along with the polymer, a suitable filler (as a pore-forming agent to control the porosity) and a plasticizer (to improve shell plasticity) were added to the shell composition. All the optimized materials (hydrophobic polymer, filler, and plasticizer) in varying compositions were mixed properly and pulverized using a mortar and pestle. A solvent system was optimized to convert the selected materials into a paste of extrudable consistency. The selected materials were mixed with a fixed volume of the solvent system to transform into a homogenous paste with continuous stirring.

### 2.3. Process Optimization for 3D Printing of Controlled Release Tablet Shells

Material for 3D printing was selected for the extrusion printing of controlled-release shells to encapsulate the tablet with an immediate release profile. Material extrusion requires optimization of various printing parameters in order to obtain stable deposition and a well-defined geometry of the controlled-release shells. These optimization parameters include consistency of the extruding paste, printing pressure, nozzle diameter (printer tip), and printing speed.

### 2.4. Design of 3D Printed Controlled-Release Tablet Shells

The geometry of controlled-release shell exploiting CAD was designed with respect to the dimensions of the selected tablet model. The design of the controlled-release shell was arranged in two parts: a shell body and a cap. The body of the controlled-release tablet shell was printed first and then the tablet was placed inside it, with subsequent printing of the cap of the shell. Different dimensions of controlled-release shells were printed to customize the drug release profile.

### 2.5. Evaluation of Propranolol HCl Tablet

#### 2.5.1. Assay/Drug Content

An assay of the propranolol HCl tablet was performed as per the method described in the British Pharmacopoeia to determine the drug content. Twenty tablets were accurately weighed and crushed into a powder form. Propranolol HCl (20 mg) was dissolved in 20 mL of distilled water and shaken for 10 min. Then, 50 mL of methanol was added to this and shaken for another 10 min. A sufficient volume of methanol (30 mL) was then added to make the volume up to 100 mL, and then filtered. Finally, 10 mL of this filtrate was diluted in 50 mL of methanol and the resulting solution was used to measure the absorbance at 290 nm. The content of propranolol HCl was then calculated as described in the Pharmacopoeia [21]. The content of propranolol HCl tablet was found to be 39.78 ± 1.14 mg, with a percentage content of 99.45% of the tablet dose.

#### 2.5.2. In Vitro Dissolution Study 

A dissolution study was carried out for the immediate-release tablet of propranolol hydrochloride using a United States Pharmacopeia (USP) type 1 dissolution apparatus in 0.1N HCl at 100 rpm. The 5-mL samples were withdrawn at regular time intervals (5, 10, 15, 20, 30, and 45 min) to quantify the drug release. The withdrawn sample was filtered through a syringe filter and spectrophotometrically analyzed to estimate the concentration of propranolol hydrochloride in release media at the wavelength of 290 nm [22].

### 2.6. Encapsulation of Propranolol HCl Tablets 

The formulated paste was placed in an extruding syringe and loaded into the 3D printer (Biobot 1). A 600-µm nozzle tip was fixed to the extruding syringe. The printing pressure was set between 40 and 60 psi based on the paste composition and consistency. The G-code files were uploaded to the printer software and the files were run to conduct the printing process. Firstly, the body of the controlled-release shell was printed; then, the propranolol HCl tablet was placed inside the printed shell. The cap of the controlled-release shell was then printed over the top of the body to close the controlled-release shell. The total printing time was approximately 4–6 min for each print. The printing speed was 4 mm/s and was kept constant. After printing, the 3D printed tablets were placed in a vacuum dryer overnight to complete the drying process. 

### 2.7. Characterization of Printed Controlled-Release Shell 

#### 2.7.1. Fourier-Transform Infrared Spectroscopy (FTIR)

FTIR was performed to detect drug–excipient interactions during the 3D printing process. Infrared spectra were obtained for the physical mixture of ingredients and the 3D printed controlled-release shell using an FTIR spectrometer (Agilent Cary630 FTIR). A small amount of powdered excipient mixture used in the shell preparation was analyzed at wavenumbers between 400 and 4000 cm^−1^. The results from the excipient physical mixture were compared to a sample of the 3D printed controlled-release shell to assess any drug–excipient interactions during the 3D printing process [23].

#### 2.7.2. Differential Scanning Calorimetry (DSC)

DSC analysis was carried out for the physical mixture of excipients (cellulose acetate, D-mannitol, and PEG 6000) and the 3D printed controlled-release shell using a TA DSC25 Instrument. The physical mixture of the material and the 3D printed controlled-release shell were powdered and sieved. An accurately weighed amount of sample (5 mg) was placed in a Tzero aluminium pan and sealed completely. The nitrogen flow rate used for the analysis was 50 mL/min and the heating rate was set at 10 °C/min using an empty Tzero aluminium pan as a reference [24].

### 2.8. In-Vitro Dissolution Study

#### 2.8.1. Drug Release Profile

In vitro drug dissolution studies were performed for the 3D printed tablets of different compositions and sizes using USP type 1 dissolution apparatus as per the monograph for propranolol HCl at 100 rpm using 900 mL of phosphate buffer (pH 6.8) as the dissolution medium at a temperature of 37 ± 0.5 °C. The amount of sample withdrawn was 5 mL at the time points of 5, 10, 15, 30, 60, 120, 240, 360, 480, 600, and 720 min. The withdrawn sample was filtered through a syringe filter and analyzed using a UV spectrophotometer at 290 nm. The dissolution studies were performed in triplicate and the cumulative percentage of drug release with respect to time was determined [25,26].

#### 2.8.2. Drug Release Kinetics

The drug release kinetics which showed the mechanism of overall release of drug from the developed formulations were subjected to quantitative and qualitative changes in formulation designs considered as rational to understand the release mechanism. The propranolol HCl release kinetics from the encapsulated tablet in the printed shells of different composition and sizes were determined by using the best-fit model [27].

## 3. Results and Discussion

### 3.1. Preparation of Material for 3D Printing of Controlled-Release Tablet Shells

Pharmaceutical product development exploiting the 3D printing technique requires specific consideration of material choice in accordance with the method used for printing. For example, the fused deposition modeling (FDM) method requires the use of thermoplastic materials extruded at high temperatures. The current investigation involved the use of a pressure-assisted microsyringe (PAM) that requires materials to be extruded at room temperature in a semi-solid state, which consequently dry after extrusion from the printer.

For the shell composition, different hydrophobic polymers were investigated in order to modify the drug release of the tablet enclosed inside the core of the shell. The material was optimized for extrusion through the 3D printer and explored for its ability to build a layer-by-layer design of controlled/sustained-release shells. The selection of the polymers and all other excipients/chemicals exploited in this investigation was based on well-reported use in literature for the preparation of tablet shells. These excipients have been well exploited using the PAM-based 3D printing technique for pharmaceutical product development [4,24,28,29] and/or the formulation of controlled/sustained-release tablets [30,31]. The polymers investigated to assess extrusion ability through the 3D printer included ethyl cellulose, hydroxyl propyl methylcellulose (HPMC), polyvinylpyrrolidone (PVP), and cellulose acetate. These polymers were dissolved in different types of solvent and tested to form a paste of desirable consistency that should be suitable for 3D printing. The solvents investigated for the formation of paste with good extrusion consistency included methanol, ethanol, isopropyl alcohol, dichloromethane, dimethyl sulfoxide, and acetone. The optimization of the solvents exploited in the preparation of a paste with good extrusion consistency should take into consideration the rheology of the formulated paste and the time required to evaporate the solvent. Solvents should not be highly evaporative, as this may cause fast drying of the extrusion material at the tip of the nozzle, ultimately causing a blockage. However, slow evaporation of the solvent may increase the time required for solidification of the printed layers and ultimately hamper the formation of the layer-by-layer 3D structure. The extrusion paste should be smooth and homogeneous in order to exhibit good flow characteristics through the nozzle tip and be free from the occurrence of large particles that may cause blockage of the nozzle tip during the extrusion process. Among the various polymers assessed for the preparation of pastes, ethylcellulose, HPMC, and PVP exhibited variable characteristics in terms of the formed paste, including consistency, rheology, and extrusion behaviour. During the deposition observation, these materials showed high wettability at the interface with the borosilicate glass substrate, which prevented the building of a layer-by-layer 3D structure. Based on the physical properties and rheological behaviour during extrusion, cellulose acetate was found to be a suitable polymer to print the controlled-release tablet shells. Polyethylene glycol (PEG) is one of the most efficient plasticizers used in polymer-based pharmaceutical product development as it has a broad range of molecular weight grades available, is nontoxic in nature, and shows miscibility with a wide range of pharmaceutical excipients as well as biodegradability [32].

Furthermore, among the different grades of PEG, the higher molecular weight grade provides a better plasticizing effect [33]; therefore, PEG 6000 was added as a plasticizer to the shell composition. The amount of solvent plays an essential role in affecting the flow behaviour of the paste. A combination of solvents was used to prepare the cellulose acetate paste for extrusion-based 3D printing. The use of a highly evaporative solvent will lead to rapid drying of the paste causing a nozzle blockage, and the use of a slowly evaporating solvent will increase the ink spreading upon printing and complicate the formation of well-defined layer-by-layer 3D structures. To overcome this, a combination of solvent mixture was used to provide the optimum rate of solvent evaporation from the paste after extrusion through the 3D printer. The miscibility/solubility profile of cellulose acetate in different solvents and the resulting solution behaviour, along with the extrudability characteristics of the paste through the 3D printer, are shown in Table 1.

The cellulose acetate was dissolved in a combination of acetone, ethanol, and DMSO in the ratio of 2.5:2.5:1 and optimized as extrusion material for the printing of controlled-release tablet shells. The DMSO added as co-solvent for cellulose acetate was within the permissible limits and concentrations safe for use in humans [34,35]. The optimized materials for the shell formation were composed of cellulose acetate, D-mannitol, and PEG 6000. It was mixed properly and pulverized using a mortar and pestle. Then, 3.0 g of the blended powder mixture of cellulose acetate, D-mannitol, and PEG 6000 was taken and mixed with a fixed volume (2.4 mL) of the solvent system (acetone, ethanol, and DMSO in the ratio of 2.5:2.5:1 v/v) to make a homogenous paste of a semi-solid consistency with continuous stirring.

### 3.2. Process Optimization for 3D Printing Controlled-Release Tablet Shells

Among the various 3D printing technologies exploited for pharmaceutical manufacturing, the extrusion-based 3D printing technique was investigated for printing controlled-release shells of the encapsulated tablet. The printer explored for the study was a pressure-assisted semi-solid extrusion-based 3D printer (BIOBOT 1). This technique was optimized for various process parameters (such as paste consistency, nozzle diameter, printing pressure, and speed) in order to print the controlled-release shell of various configurations. Firstly, the paste was formed, which was well extruded through the printer nozzle. The cellulose acetate was dissolved in a combination of acetone, ethanol, and DMSO in a ratio of 2.5:2.5:1, along with a suitable filler (D-mannitol) and plasticizer (PEG 6000) in order to print a stable layer of controlled-release shell wall with the optimum hardness and drying time. The prepared paste was placed in an extrusion syringe with nozzles of different diameters attached to the syringe for printing. The nozzle diameter was optimized for printing. The selection of nozzle size was based on the consistency, flowability, and particle size of the paste. Tapered nozzles with inner tip diameters of 400–600 µm were optimized for printing the controlled-release shells. The printing pressure and printing speed were optimized based on the consistency of the paste. The printing pressure influences the flow behaviour of the paste through the nozzle and relies on the composition of the paste. The printing pressure was varied based on the amount of filler added to the composition of extruding paste; 40–60 psi was found to be the optimum pressure for the printing of controlled-release shells. Similarly, the printing speed of 4 mm/s was found to be optimum for printing controlled-release shells. All the printing parameters were kept constant throughout the printing process; the optimization results are summarized in Table 2.

### 3.3. Design of 3D Printed Controlled-Release Tablet Shells

**Figure 1:**
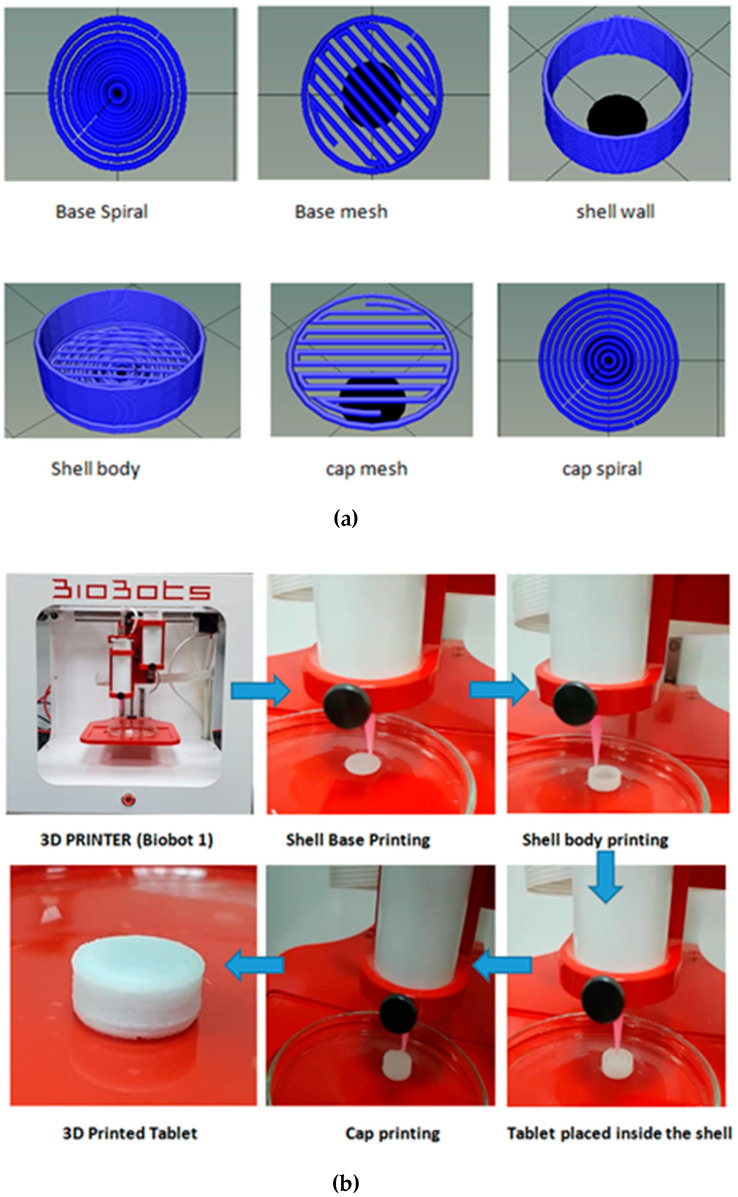
(**a**) Stereolithography (STL) files illustrating the design of shell body/wall and cap. (**b**) The printing process is enclosing the marketed tablet inside the 3D printed controlled/sustained-release shell.

The empty shell shape was designed to be round, similar to the shape of the tablet chosen for the study. The size of the selected tablet was 8 mm in diameter and 2.6 mm in thickness. Controlled release shells of three different sizes (i.e., fixed, medium, and large) were designed to demonstrate the influence of the gap/space between the shell wall and the enclosed tablet on the dissolution time and drug release behaviour. The optimized design for printing the tablet shells was developed as a stereolithography (STL) file and ultimately sliced through a Repetier-Host to create G-code to print the controlled-release shell for the tablet (illustrated in Figure 1a). 

### 3.4. Printing of Controlled-Release Shells to Enclose Propranolol Hcl Tablets

After the optimization of process parameters for 3D printing of the controlled-release shell, cellulose acetate along with D-mannitol and PEG 6000 were converted into a paste by dissolving them in a combination of acetone, ethanol, and DMSO at a ratio of 2.5:2.5:1. The paste was placed into the extruding syringe immediately to avoid unnecessary loss of solvent that could cause drying of paste and influence its consistency. During the filling of paste into extruding syringe, care should be taken to avoid air bubble entrapment as its presence may influence the relaxation behavior of the paste. The air bubbles were minimized by transferring the paste from an initially filled syringe to another syringe with a seal placed near the barrel tip by using a syringe connector. The printing was done on a glass substrate. Once the body of the shell was printed, the marketed tablet was placed inside the shell with the use of tweezers, and then the cap was printed over the top of the enclosed tablet. The design geometry of the cap and base was identical to provide a uniform and controlled/sustained release of propranolol HCl from the developed formulation system. The first layer of the cap design was a mesh pattern to help the tablet remain intact in the center of the shell, whereas the second layer of the cap was printed over the first layer in a spiral pattern (illustrated in Figure 1b). This printed formulation system enclosing the tablet was then tested to provide a proof of concept for the 3D printing technique used to modify the drug release profile of an immediate-release tablet.

### 3.5. Characterization of 3D Printed Controlled-Release Shells Enclosing the Immediate-Release Tablets

**Figure 2:**
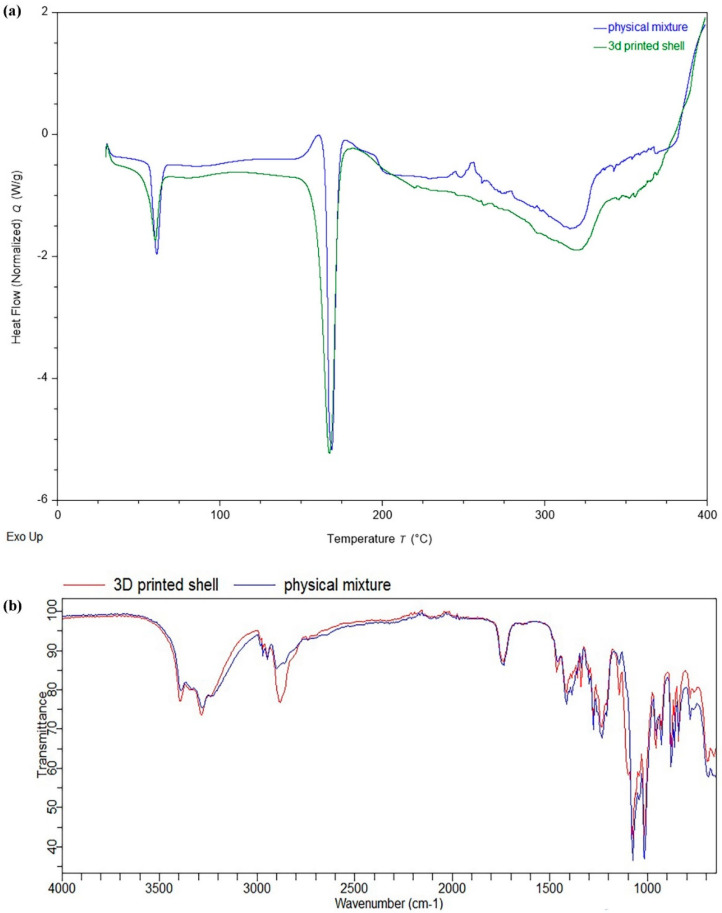
(**a**) Differential scanning calorimetry (DSC) thermogram of a physical mixture of excipients and the 3D printed shell; (**b**) Fourier-transform infrared spectroscopy (FTIR) spectra of a physical mixture of excipients and the 3D printed shell.

FTIR and DSC analysis were performed in order to determine the potential impact of various unit operations such as grinding, mixing, solvent addition, paste formation, and drying involved during the 3D printing process on the material’s behaviour. Furthermore, possible interactions between the excipients during formulation development were also assessed. The DSC thermogram of the physical mixture containing PEG 6000, D-mannitol, and cellulose acetate exhibited endothermal peaks (melting point) at 61.22 °C, 168.94 °C, and 321.13 °C, respectively, while the 3D printed shell showed no significant changes in the endothermic peaks compared to the excipients used in its formulation (Figure 2a). This reveals that there were no significant interactions between formulation excipients during the printing process. Similarly, the FTIR spectra of the physical mixture of the materials (PEG 6000, D-mannitol, and cellulose acetate) and the 3D printed shell did not exhibit any significant shift in the position of the characteristic peak (Figure 2b). The FTIR and DSC results indicate that there were no significant interactions between formulation excipients during the 3D printing process.

### 3.6. In Vitro Dissolution Study

#### 3.6.1. Influence of the Composition of 3D Printed Controlled-Release Shells

**Figure 3:**
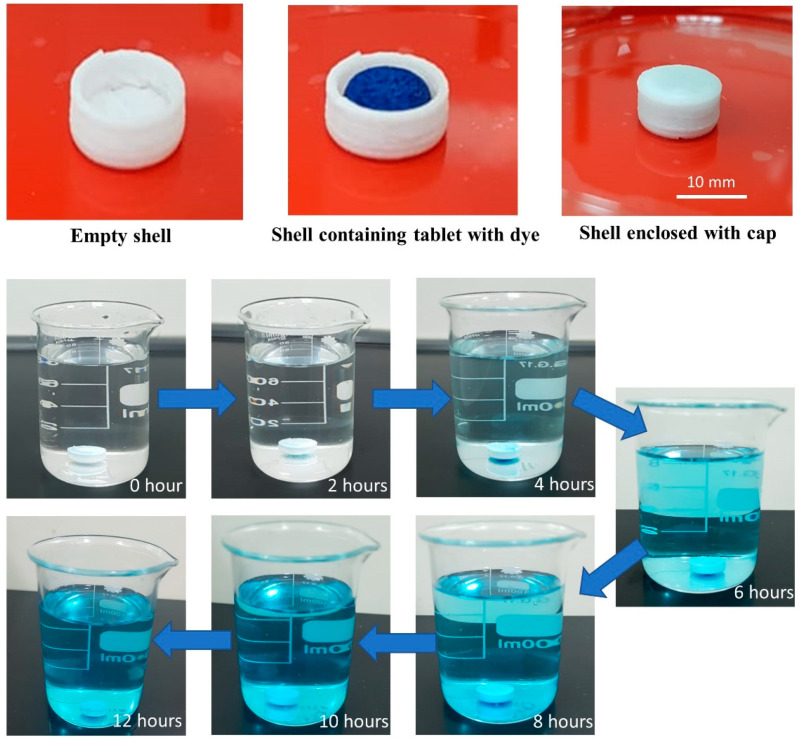
Illustration representing encapsulation of the propranolol HCl tablet soaked with color dye (methylene blue) kept inside 3D printed controlled-release shell, with the gradual increase in the intensity of the color solution showing the visual observation of methylene blue release through the 3D printed controlled-release shell at different time intervals.

Before performing the dissolution studies of the enclosed tablet, the sealing of the shell was investigated. The tablet model was soaked with color dye (methylene blue) and enclosed inside the 3D printed controlled-release shell. Then, the enclosed shell was placed in a 100-mL beaker filled with distilled water. The visual observation of the gradual change of the color intensity of the water by the methylene blue at a different time intervals (Figure 3) proves that there was no sudden leakage of the tablet content from the shell, and that the release of methylene blue through the shell was controlled.

**Figure 4:**
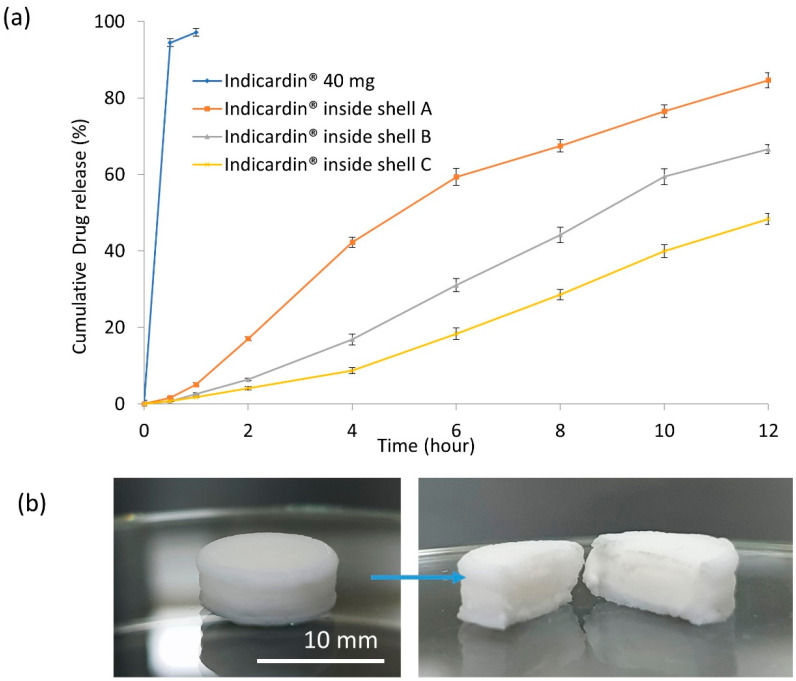
(**a**) Cumulative release of propranolol HCl from the model tablet with phosphate buffer (pH 6.8) as dissolution media at temperature 37 ± 0.5 °C versus when enclosed inside 3D printed coating shells with different excipient ratios (Table 3). (**b**) The coating shell system still intact after the dissolution experiment. The cross-section of the shell shows the expansion of the propranolol HCl tablet inside the shell.

The in vitro dissolution study was carried out to determine the release of propranolol HCl from the developed 3D printed shell and the encapsulated tablet. The dissolution study of the tablet model revealed an immediate release profile of propranolol HCl, with >95% of the drug released within 20 min. It was found that the release of propranolol HCl from the 3D printed shell showed controlled/sustained release compared to the tablet model (Figure 4a). This result validates the proof of concept that the 3D printing technique has potential applications in modifying the drug release profile of conventional immediate-release tablets. 

The composition of the controlled-release shells (shells A, B, and C), as shown in Table 3, influenced the release profile of propranolol HCl (Figure 4a). Among the three different shell formulations, shell A exhibited 84% propranolol release while shell B and C allowed for 66% and 48% release of propranolol HCl after 12 h, respectively. Figure 4b showed the intact shell after the 12-h dissolution time, and the cross-section of the shell system showed the expansion of the disintegrated tablet inside the shell without breaking it during the dissolution.

The alteration in release profiles of propranolol HCl was due to the variability in the amount of rate-controlling polymer (cellulose acetate) and the pore-forming agent (D-mannitol) used in the formulation composition. The results demonstrate that an increase in the amount of D-mannitol in the shell composition led to an increase in drug release. This behaviour is correlated to the micropores formed by the rapid dissolution of D-mannitol in the controlled-release shell, which ultimately enhances the water intake, driven by the difference in the osmotic pressure across the shell. This facilitates faster dissolution of the enclosed tablet compared to the shell composition with less D-mannitol. Khaled et al. also reported the impact of variable amounts of cellulose acetate as a drug release retardant in their investigations [4,28].

#### 3.6.2. Influence of the Size of the 3D Printed Controlled-Release Shell 

**Figure 5:**
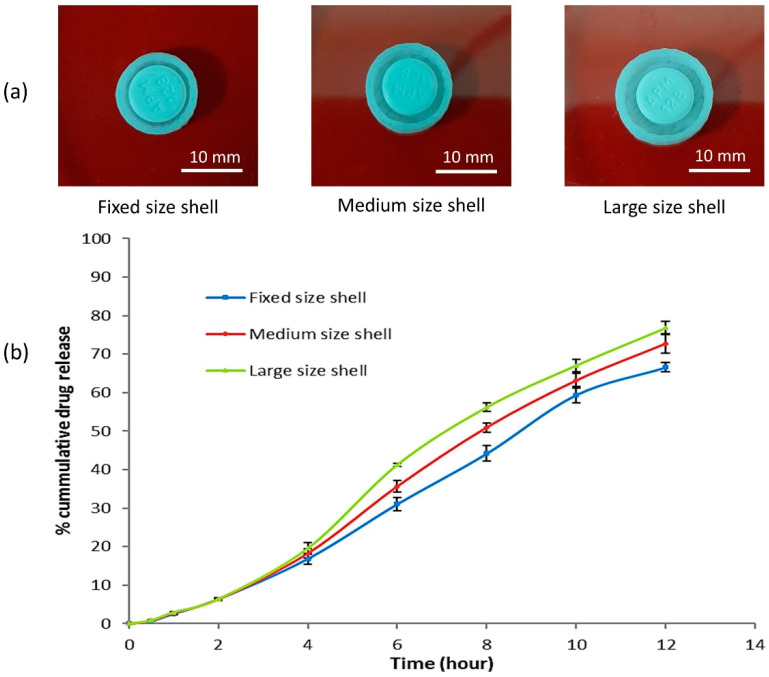
(**a**) Different sizes of 3D printed controlled-release shells illustrating the gap/space between the enclosed tablet and the shell wall. (**b**) Cumulative release of propranolol HCl with phosphate buffer (pH 6.8) as dissolution media at temperature 37 ± 0.5 °C from 3D printed controlled-release shells of different sizes.

The formulation of shell B had a balanced ratio between cellulose acetate and D-mannitol (Table 3). Therefore, it was selected to assess further the influence of different sizes of 3D printed coating shells on the cumulative release of the propranolol HCl. Three different-sized shells were printed (fixed, medium, and large). The outer and inner diameter and the gap between the shell wall and the tablet are shown in Table 4. The uncapped shell and the enclosed tablet of the three shell sizes are shown in Figure 5a. The in vitro dissolution work was carried out to evaluate the propranolol HCl release from the enclosed tablet inside the three different shells varying in sizes (Figure 5b). The propranolol HCl release rate was enhanced with respect to an increase in the size of the shell (Figure 5b).

Over a period of 12 h, 66.60%, 72.68%, and 76.81% of propranolol HCl was released from the tablet enclosed in the fixed-, medium-, and large-sized shells, respectively (Table 4). As the space between the shell wall and the tablet increased, so did the amount of dissolution medium that filled that space, and consequently, there was more dissolved propranolol HCl from the enclosed tablet. The increase in the shell size provided a larger area (space/gap between the shell wall and the enclosed tablet) available for the tablet to expand during the disintegration process, resulting in more dissolution and drug release (Figure 5a,b). The concept of accommodating the space/gap between the enclosed tablet and the shell wall demonstrated a novel idea, paving the way to explore a different approach and hypothesis. The provided space/gap could be utilized for filling with release enhancers/solubilizers or release retardant, and the drug release from the enclosed tablet might be customized as per desirability into a delayed-release or time-dependent drug release system.

#### 3.6.3. Drug Release Kinetics

Kinetic modeling plays an essential role in determining the drug release mechanism [36,37]. The drug release data were fitted by using the zero-order, first order, Higuchi, and Korsmeyer–Peppas models to investigate the best-fit model [36,38]. The results are shown in Table 5. 

Diffusion-based drug release can be either Fickian or non-Fickian [39]. In Fickian diffusion, the release rate is independent of the drug concentration in the dosage forms. The zero-order release kinetics model describes delivery systems where the drug release rate is constant with respect to time. In non-Fickian diffusion, several factors can affect the drug release, and based on these factors, the release can be predicted by several mathematical models like the first-order release kinetics model, the Higuchi release kinetics model, and the Korsmeyer–Peppas release kinetics model, etc. In first-order release kinetics, the drug release rate is concentration-dependent. The Korsmeyer–Peppas release kinetic model relates the drug release exponentially to the fractional release of the drug [40]. The equation can be written as follows:

*Qt/ Q∞* = *kt^n^*

where *Qt/ Q∞* is a fraction of drug released at time *t*; *k* is the release rate constant; and *n* is the release exponent.

The value of *n* can indicate likely mechanisms for drug release. For example, Ritger and Peppas determined that *n* should be in the range of 0.425–0.500 for disk-shaped devices if the release profile indicates that diffusion of dissolved drug through a matrix is the dominant release mechanism [41,42]. The *n* value of about 1.0 indicates that polymer relaxation, polymer dissolution, or tablet erosion is the dominant mechanism. An intermediate value suggests a combination of mechanisms is effective, involving the so-called non-Fickian diffusion, anomalous release, or mixed transport.

Among the shell compositions investigated, the release of propranolol HCl from shell A (containing 20% cellulose acetate and 65% mannitol) was best fitted to first-order kinetics, with an R^2^ value of 0.9939, showing that the release rate depends on its concentration. The release of propranolol HCl from shells B and C (containing 40% and 60% cellulose acetate and 45% and 25% mannitol, respectively) was best fitted to the Korsmeyer–Peppas model, with R^2^ values of 0.9954 and 0.9959, respectively. n-Values ranged between 0.576 and 0.619, indicating a non-Fickian diffusion model of drug release.

Similarly, the release of propranolol HCl from the tablets enclosed in shells (the composition of formulation B), including fixed-, medium- and large-sized shells, was best fitted to the Korsmeyer–Peppas model, with R^2^ values of 0.9954, 0.9964, and 0.9942, respectively. Shell sizes with fixed-, medium-, and large geometry showed n-values of 0.619, 0.621, and 0.622, respectively. This indicates a non-Fickian diffusion model of drug release. Among the three geometries, the fixed size was more compact; therefore, less area was available for exposure to the dissolution media and hence the release rate was lower compared to drug release from the medium and the large shell. In contrast, the medium and large sizes of the shell provided more area for exposure to the dissolution media, leading to faster disintegration of the tablet and thus an improved release rate of propranolol HCl.

The current approach shows that the modulation of the shell formulation and size represents an opportunity to control the amount and rate of propranolol HCl released from the off-the-shelf tablet. This novel idea can be developed further and applied to encapsulate the available conventional dosage forms to fulfil the needs of personalized medicine. This new perspective on the use of 3D printing could be applied to customize the amount and release profile of the enclosed available conventional drug tablet instead of developing a 3D printing model for each drug.

Promising dosage forms with modified-release profiles as per desirability have been fabricated using 3D printing technology [6]. However, the content verification, the formulation supply of each drug, and the expected competition between the conventional and the 3D printed dosage forms of the same drug are among the various difficulties found in the application of 3D printing in the healthcare system [8]. Here, we suggest that the encapsulation of conventional immediate-release tablets using 3D printed coating shells will bridge the gap between the currently available conventional tableting methods and the future applications of 3D printing technology to meet the final goal, which is to provide effective treatment with minimum adverse effects.

## 4. Conclusions

The fabrication of an encapsulating shell using extrusion-based 3D printing at room temperature showed no significant impact on the physicochemical character of the materials used to design the encapsulating system. This method provided the ability to use pharmaceutical grade excipients in different ratios without affecting the quality of the printed product. The use of different ratios of cellulose acetate to D-mannitol for the fabrication of the encapsulating shell showed the ability to control the amount and the rate of propranolol HCl released from off-the-shelf tablet (Indicardin^®^). Further, the dissolution profile of the 3D printed controlled-release shell of different sizes also influenced the release of propranolol HCl, in which the combination of material composition and shell size allowed for a fine-tuning of the release profile. This approach could be explored further to help healthcare professionals customize the drug amount and the release profile of the available conventional tablets to provide the greatest therapeutic efficacy with the minimum untoward effects commonly associated with the administration of the immediate-release tablets.

## Figures and Tables

**Table 1:** Miscibility/solubility profile of cellulose acetate in different solvents and the resulting solution behaviour, along with extrudability characteristics of the paste through the 3D printer.

Solvent	Solubility	Solution Behavior and Extrudability Through the 3D Printer
Water	Insoluble	Insoluble mass
Water with surfactant (Tween 80)	Insoluble	Granular mass
Isopropyl alcohol	Insoluble	Insoluble mass
Ethanol	Insoluble	Insoluble mass
Dichloromethane	Insoluble	Insoluble mass
Ethyl acetate	Partially soluble	Segregated mass
Acetone	Soluble	Paste extruded rapidly, but tip blockage occurred due to drying of the paste over time
Acetone/water (1:1 ratio)	Insoluble	Segregated mass
Acetone/ethanol (1:1 ratio)	Insoluble	Gummy mass
Acetone/isopropyl alcohol (1:1 ratio)	Insoluble	Gummy mass
Acetone/propylene glycol (4:1 ratio)	Soluble	Paste but hindered extrusion
Acetone/isopropyl alcohol (3:2 ratio)	Soluble	Paste but no continuous extrusion
Acetone/ethanol/ dimethyl sulfoxide (DMSO) (2.5:2.5:1 ratio)	Soluble	Good uniformity and continuously extrudable paste

**Table 2:** Optimization for the extrusion-based 3D printing process for printing controlled-release tablet shells.

Process Parameters	Value	Outcome/Observation
Nozzle size (µm)	200–400 µm	No extrusion
	400–600 µm	Good extrusion with an optimum layer thickness
	600–800 µm	Very rapid extrusion with an increased layer thickness
Extrusion pressure (psi)	20–40 psi	No extrusion
	40–60 psi	Good extrusion with an optimum layer thickness
	60–80 psi	Very rapid extrusion with an increased layer thickness
Printing speed (mm/s)	2 mm/s	Slow printing
	4 mm/s	Optimum printing
	6 mm/s	Rapid printing, sagging due to wetting
Nozzle shape	Blunt tip	Tip blockage and hindered extrusion
	Tapered tip	Smooth and continuous extrusion

**Table 3:** Influence of different compositions of 3D printed controlled-release shell on % cumulative drug release after 12 h with phosphate buffer (pH 6.8) as dissolution media at a temperature of 37 ± 0.5 °C.

Formulation	% Composition Cellulose Acetate D–Mannitol PEG 6000			% Cumulative Drug Release After 12 h
Shell A	20	65	15	84.64 ± 1.99
Shell B	40	45	15	66.60 ± 1.13
Shell C	60	25	15	48.37 ± 1.46

**Table 4:** Influence of different sizes of 3D printed controlled-release shells on cumulative drug release after 12 h with phosphate buffer (pH 6.8) as dissolution media at temperature 37 ± 0.5 °C.

Shell Size	Shell Dimensions			% Cumulative Drug Release After 12 h
	Shell Outer Diameter	Shell Inner Diameter	The Gap between the Shell Wall and Tablet	
Fixed-sized shell	12 mm	9.6 mm	0.8 mm	66.60 ± 1.13
Medium-sized shell	13 mm	10.6 mm	1.3 mm	72.68 ± 2.34
Large-sized shell	14 mm	11.6 mm	1.8 mm	76.81 ± 1.60

**Table 5:** In vitro release kinetics of propranolol HCl from 3D printed controlled-release shells of variable composition and size fitted to the best-fit release kinetic model.

Formulation Type	Zero Order (r^2^)	First Order (r^2^)	Higuchi (r^2^)	Korsmeyer–Peppas	
				(r^2^)	*n* Value
**Composition-based**					
Formulation A	0.9521	0.9939	0.9906	0.9662	0.542
Formulation B	0.9937	0.9741	0.9503	0.9954	0.619
Formulation C	0.9817	0.9541	0.9106	0.9959	0.576
**Size-based**					
Fixed-sized shell	0.9937	0.9741	0.9503	0.9954	0.619
Medium-sized shell	0.9949	0.9622	0.9516	0.9964	0.621
Large-sized shell	0.9902	0.9583	0.9583	0.9942	0.622
